# Identification of Synaptic Targets of *Drosophila* Pumilio

**DOI:** 10.1371/journal.pcbi.1000026

**Published:** 2008-02-29

**Authors:** Gengxin Chen, Wanhe Li, Qing-Shuo Zhang, Michael Regulski, Nishi Sinha, Jody Barditch, Tim Tully, Adrian R. Krainer, Michael Q. Zhang, Josh Dubnau

**Affiliations:** 1 Cold Spring Harbor Laboratory, Cold Spring Harbor, New York, United States of America; 2 Graduate Program in Molecular and Cellular Biology, State University of New York Stony Brook, Stony Brook, New York, United States of America; Columbia University, United States of America

## Abstract

*Drosophila* Pumilio (Pum) protein is a translational regulator involved in embryonic patterning and germline development. Recent findings demonstrate that Pum also plays an important role in the nervous system, both at the neuromuscular junction (NMJ) and in long-term memory formation. In neurons, Pum appears to play a role in homeostatic control of excitability via down regulation of *para*, a voltage gated sodium channel, and may more generally modulate local protein synthesis in neurons via translational repression of *eIF-4E*. Aside from these, the biologically relevant targets of Pum in the nervous system remain largely unknown. We hypothesized that Pum might play a role in regulating the local translation underlying synapse-specific modifications during memory formation. To identify relevant translational targets, we used an informatics approach to predict Pum targets among mRNAs whose products have synaptic localization. We then used both in vitro binding and two in vivo assays to functionally confirm the fidelity of this informatics screening method. We find that Pum strongly and specifically binds to RNA sequences in the 3′UTR of four of the predicted target genes, demonstrating the validity of our method. We then demonstrate that one of these predicted target sequences, in the 3′UTR of *discs large (dlg1)*, the *Drosophila* PSD95 ortholog, can functionally substitute for a canonical NRE (Nanos response element) in vivo in a heterologous functional assay. Finally, we show that the endogenous *dlg1* mRNA can be regulated by Pumilio in a neuronal context, the adult mushroom bodies (MB), which is an anatomical site of memory storage.

## Author Summary

The *Drosophila* Pumilio (Pum) protein was originally identified as a translational control factor for embryo patterning. Subsequent studies have identified Pum's role in multiple biological processes, including the maintenance of germline stem cell, the proliferation and migration of primordial germ cells, olfactory leaning and memory, and synaptic plasticity. Pum is highly conserved across phyla, i.e., from worm to human; however, the mRNA targets of Pum within each tissue and organism are largely unknown. On the other hand, the prediction of RNA binding sites remains a hard question in the computational field. We were interested in finding Pum targets in the nervous system using fruit flies as a model organism. To accomplish this, we used the few Pum binding sequences that had previously been shown in vivo as “training sequences” to construct bioinformatic models of the Pum binding site. We then predicted a few Pum mRNA targets among the genes known to function in neuronal synapses. We then used a combination of “golden standards” to verify these predictions: a biochemical assay called gel shifts, and in vivo functional assays both in embryo and neurons. With these approaches, we successfully confirmed one of the targets as Dlg, which is the *Drosophila* ortholog of human PSD95. Therefore, we present a complete story from computational study to real biological functions.

## Introduction

*Drosophila melanogaster* Pumilio (Pum) protein is one of the founding members of the PUF RNA-binding protein family. Its function in the posterior body patterning of *Drosophila* embryos is relatively well studied. Wharton and Struhl [1] first identified two copies of sequence elements located in the 3′ untranslated region (3′UTR) of maternal *hunchback* (*hb*) mRNA, named Nanos Response Elements (NREs), which are essential for normal abdominal segmentation. It was later shown that Pum binds these elements, recruits Nanos (Nos) and Brain Tumor (Brat), and represses the translation of maternal *hb* mRNA [2]. Pum was also reported to temporally regulate the translation of *Drosophila bicoid* (*bcd*) mRNA, which plays a key role in anterior development [3]. In addition, Pum, acting together with Nos, is required for germline development in *Drosophila* embryos, and *Cyclin B* (*CycB*) mRNA appears to be a target of translational repression by this complex [4],[5]. As a characteristic of the PUF family proteins, the minimal RNA-binding domain of Pum comprises eight imperfect repeats, is evolutionarily conserved across species from yeast to human [6] and, therefore, is termed the PUF domain or Pumilio Homology Domain (Pum-HD). This RNA-binding domain appears to be sufficient for the function of Pum in vivo during *Drosophila* abdominal segmentation [7].

More recently, Pum has been found to play a role in the nervous system at the neuromuscular junction [8]–[11], in voltage-gated Na^+^ current homeostasis in the CNS [9] and in long-term memory [12]. Dubnau et al. [12] employed the complementary “genomics” approaches of (i) a large-scale behavioral screen for mutants defective in one-day memory, and (ii) DNA microarray screening to identify genes in normal flies that are transcriptionally regulated during long-term memory formation. *pum* was found with both approaches: it is transcriptionally upregulated during memory formation after spaced training (which results in long-term memory) relative to massed training (which results only in shorter forms of memory), and two independent transposon insertions into *pum* yielded mutants with defective one-day memory after spaced training. In addition to *pum*, six other components of a pathway putatively involved in local translational control were identified: *staufen*, *orb* (CPEB), *moesin* and *eIF-2G* were transcriptionally regulated during memory formation, whereas transposon-mediated lesions were found in or near *oskar* (*norka* mutant) and *eIF-5C* (*krasavietz* mutant).

Local mRNA translation within dendrites of neurons has been proposed to be a mechanism for activity-dependent synaptic plasticity (reviewed in [13]). We hypothesized that Pum might play a role in local translation involved in synapse-specific modifications during memory formation [12]. Consistent with this notion, Ye et al. [10] showed that Pum and Nos act together and play a critical role in the morphogenesis of high-order dendritic branches in *Drosophila* peripheral neurons, and that Nos colocalizes with RNA granules in dendrites. The role of Pum-dependent regulation in neurons also may be conserved [14].

Despite these genetic observations of Pum/Nos function in neurons, only a few neuronal targets of Pum have been demonstrated in vivo [8],[9]. A large number of Pum-associated mRNAs have been recently identified from oocytes and early embryos [15]. These include a number of neuronally expressed genes whose in vivo relationship with Pum remains to be shown. As a complementary approach to screen for potentially relevant neuronal (and in particular synaptic) targets of Pum, we have used a combination of informatics and experimental approaches. Our first step to identify new Pum targets was to characterize and model the Pum binding sites. We then used our models to predict the presence of NREs in the 3′UTRs of mRNAs coding for synaptic proteins. We validated several of these by in vitro binding assays. We then used an established in vivo functional assay [1] to demonstrate Pum-dependent repression via the predicted NRE in the 3′UTR of *dlg1*. Finally we demonstrated that transgenic over-expression of Pum is sufficient to reduce endogenous levels of Dlg protein in Kenyon cell neurons of the mushroom body.

## Results

**Figure 1:**
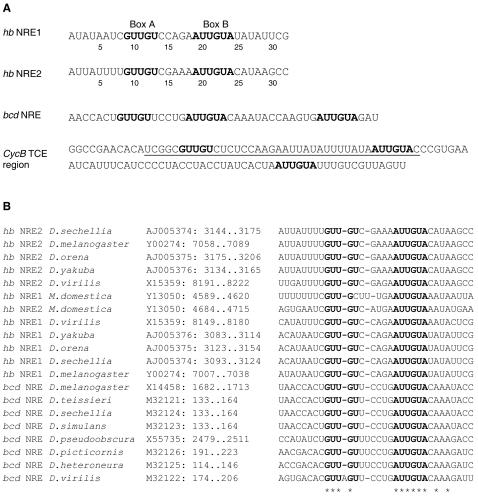
Sequences of Known NREs and Their Conservation Across Fly Species. (A) Known NREs in *hb* and *bcd* as identified by Wharton and Struhl [1] and *CycB* TCE (with flanking sequences) by Dalby and Glover [22]. Deletion of the underlined sequence in the *CycB* 3′UTR disrupts translation control. Conserved boxes in these sequences are in boldface. (B) Alignment of NREs in *hb* and *bcd* across several fly species. On the left are the gene names and species. In the middle are the Genbank accessions and the positions of the sequence segments shown in the alignment on the right. Box A and Box B are highlighted in bold face. Sequence alignment columns that are completely conserved are labeled with asterisks.

As a first step to predict novel Pum targets, we attempted to model the known NREs. The known targets of Pum include *hb*, *bcd*, *CycB* and *eIF-4E*. At the time we initiated this study, the exact binding sites of Pum on *eIF-4E* and *CycB* were unclear [5]. In contrast, the NREs in *hb* and *bcd* mRNAs are relatively well studied, with both in vitro binding assays and in vivo functional tests on wild-type and mutated sites [1],[2],[6],[7],[16]. The *hb* transcript contains two NREs and the *bcd* mRNA contains one copy of the NRE that is very similar to *hb* NREs (Figure 1).

As defined by Wharton and Struhl [1], the NREs are 32-nucleotide sequences with two “boxes” that are well conserved across sites and between fly species (Figure 1). The two conserved boxes are often referred as Box A and Box B. Mutation and footprinting studies on *hb* NRE2 suggested that a GST-fused Pum RNA-binding domain contacts both Box A and Box B [2],[7], consistent with the binding results by Zamore et al. [6] on a 37-nt RNA sequence comprising one *hb* NRE and mutants thereof. Particularly, mutations in the last four nucleotides of Box B (UGUA, base 21–24, Figure 1A) appear to have the strongest effects on both Pum binding and in vivo function of the NRE, whereas many mutations around Box A appear to have weaker effects [7]. *hb* NRE bases 17–20 appear to be important for Pum to recruit Nos but not for Pum binding per se [7],[16]. Recent results from a structural study of human pumilio-homology domain [17], a study of binding specificity and mRNA targets of a *C. elegans* PUF protein [18] and a genome-wide identification of mRNAs associated with *Drosophilia* Pumilio [15], strongly indicate that Box B sequences are crucial for binding to Puf proteins. In the case of *bcd*, which contains a single NRE, there is a half-site shortly downstream containing Box B. The NRE-related boxes in *bcd* and *hb* are evolutionarily conserved across several *Drosophila* species (Figure 1B and [3],[19]). We used these NREs, and their conservation, as starting points to better define a model for NRE prediction.

### NRE Models for Fly Species

**Figure 2:**
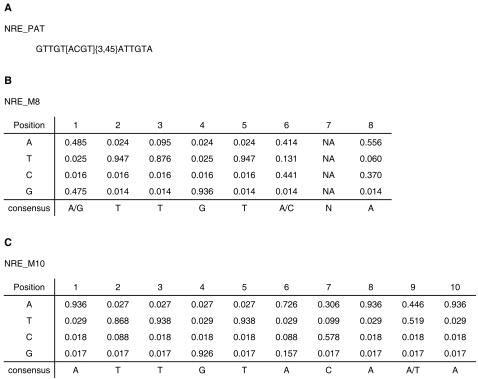
Models of NREs Constructed To Predict New Putative Pum-Binding Sites. (A) Sequence pattern as a regular expression. [ACGT] {3, 45} matches any sequences from length 3 to 45. (B,C) Base-frequency matrices obtained using Gibbs Sampler with different parameter settings. Position 7 of NRE_M8 is a motif gap, which means the base in this position is irrelevant. Here we use DNA notation instead of RNA notation because the transcript sequences from the genome project are in DNA notation.

We constructed three alternative models for Pum-binding sites, based on different assumptions. The first model is a simple consensus pattern, and the other two are based on positional weight matrices (PWM) [20],[21]. The three alternative models of the NRE are shown in Figure 2. NRE_PAT is a simple consensus of known NREs in *hb* and *bcd,* and translational control element (TCE) in *CycB* [22]. The conserved boxes in NREs suggest a pattern in which Box A precedes Box B. Both of the boxes may be important as previous studies suggested that Pum makes contact with both of them [2],[6],[7]. The distance between Box A and Box B in *bcd* of some fly species is one base longer than *melanogaster*, suggesting that the distance between the two boxes may be flexible. *CycB* TCE also contains short sequence segments like Box A and Box B and the distance between them is 23 bp. Therefore, we arbitrarily set the distance between 3 to 45 bases, to reduce the chance of missing some possible functional sites.

NRE_M8 and NRE_M10 are frequency matrices generated by Gibbs Sampler (see Materials and Methods). NRE_M8 is based on the assumption that both Box A and Box B may bind Pum. We used the recursive mode of Gibbs Sampler to require each sequence to contain two to three binding sites. In the output, the program actually picked two sites in each sequence. When we required a longer motif length, the information content at the additional position was very low. Therefore, we stopped at this motif with seven valid positions and one gap. NRE_M10 is based on the assumption that only Box B is important for Pum binding. This was derived from the data of the human Pum-RNA crystal structure [17]. We used the site sampler mode of the Gibbs program, which assumes that each sequence contains exactly one binding site. We picked the motif length 10 because this motif happened to cover Box B and four bases downstream, which contact Pum in the crystal structure. It is also worth noting that two of these four downstream bases are conserved across fly species in *hb* and *bcd* NREs (Figure 1B).

### Prediction of New Pum Targets in Synaptic Genes

The predicted Pum targets among the 151 synaptic genes with the above three NRE models are listed in Tables S1, S2, S3, respectively. With NRE_PAT, only five transcripts/genes are predicted. Among them, the pattern match is conserved between *D. melanogaster* and *D. pseudoobscura* in only one gene, *dlg1* (transcript isoforms A and D, which contain identical 3′UTRs). Conservation is unknown for two genes, *AP-1 gamma* and *mam*, because corresponding *D. pseudoobscura* 3′UTR sequences were not available at the time we initiated this study. Non-conserved predictions on *CaMKII* and *EP2237* could be false positives. However, it is also possible that the 3′UTR sequences of those two genes are incomplete or inaccurate for *D. pseudoobscura* (at the time we initiate this study), resulting in the failure to find conserved sites.

With NRE_M8, 31 transcripts (28 genes) are predicted to be candidate Pum targets. Here, we require that a 3′UTR sequence must contain at least two high-score sites to be considered as a candidate Pum target. Among them, 10 transcripts (8 genes) have at least two predicted sites that are conserved between the two fly species. With NRE_M10, 28 transcripts (25 genes) are predicted to be candidate Pum targets, among which 11 transcripts (9 genes) have at least one conserved site. Notably, *dlg1* gene is predicted to be a candidate Pum target all three NRE models. In addition, the target *dlg1* has also been previously suggested as a potential Pum target based on its presence in a collection of 1434 *Drosophila* genes containing the motif UGUAHAUA [15].

### In Vitro Validation of Predicted Pum Targets

Candidate memory genes from our previous studies [12] were sorted by their effect size (i.e., differential expression in microarray experiments). 12 transcripts (11 genes) with predicted Pum binding sites were chosen for further testing based on their ranking in the candidate memory gene list and their relevance to memory and/or synaptic functions as annotated in FlyBase. Among those, we successfully obtained the 3′UTR sequence segments in 9 transcripts by PCR, to make templates for in vitro transcription. These target genes, their Pum-binding predictions, and the locations of tested 3′UTR segments are listed in Table 1. Among these, the *dlg1* gene has predicted Pum binding sites in the 3′UTR of two non-overlapping transcript isoforms (also refer to Figure S4).

**Table 1:** Genes (Transcripts) Selected for Electrophoretic Mobility Shift Assay (EMSA)

Gene Description			Predictions			EMSA Results		
Gene Symbol	FlyBase ID	RepresentativeTranscript	NRE_PAT Match	NRE_M8 (cutoff = 7.5, max = 10.97)	NRE_M10 (cutoff = 10, max = 15.21)	Probe Position in 3UTR	Percent Binding	Binding Sites
*Ace*	FBgn0000024	CG17907-RA		9.119 (751), 8.875 (814)^a^, 9.798 (1265)^o^, 9.119 (1351)	14.78 (814)^a^, 10.34 (1229)^a^	722–1095	33	1
*AP-1gamma*	FBgn0030089	CG9113-RA	122–141 (GTTGT..9..ATTGTA)^n^	10.97 (122)^n^, 8.873 (136)^n^, 8.349 (240)^n^		85–191	21.8	1
*Csp*	FBgn0004179	CG6395-RA		7.666 (109)^a^, 8.872 (1050), 7.666 (1242)^a^, 9.795 (1303), 9.119 (1519)^a^, 7.666 (1561), 7.666 (1909)	13.95 (87), 10.13 (1537)^a^	1218–1545	57.2	2
*dlg1*	FBgn0001624	CG1725-RA	1491–1534 (GTTGT..33..ATTGTA)^a^	9.8 (367)^a^, 10.97 (1491)^a^, 8.872 (1529)^a^	12.57 (367)^a^	1485–1539	56.9	1
*dlg1*	FBgn0001624	CG1725-RC		8.59 (277), 8.59 (783), 10.04 (793)		744–829	0	0
*EP2237*	FBgn0043364	CG4427-RA	401–443 (GTTGT..32..ATTGTA)	9.12 (390)^a^	10.40 (318)	340–514	20.5	1
*Gad1*	FBgn0004516	CG14994-RA		8.872 (730), 8.595 (950)		704–973	35.9	2
*mam*	FBgn0002643	CG8118-RA	716–735 (GTTGT..9..ATTGTA)^n^	9.795 (7)^n^, 9.8 (716)^n^, 8.875 (1037)^n^	10.40 (1037)^n^	642–1110	73.8	2
*shn*	FBgn0003396	CG7734-RA		8.872 (480), 7.666 (835), 7.72 (913)^a^, 8.875 (983)^a^, 8.873 (1105)^a^	10.78 (895)^a^, 14.78 (983)^a^	809–1013	59	2

Gene symbols, FlyBase IDs, and transcript IDs are from the Berkeley *Drosophila* Genome Project (BDGP) Release 3.1 annotation. Pattern-match predictions are represented as: *start-end coordinates (matched sequence).* The number between the dots represents the length of the sequence between the two boxes. Matrix predictions are represented as: *score(coordinate).* The coordinate corresponds to the first position in the matrix. The probe positions are shown as a coordinate range. All coordinates are referred to the stop codon (the first nucleotide of the stop codon as coordinate 1).

Superscripts in the predictions represent the conservation between *D. melanogaster* and *D. pseudoobscura*.

a The predicted site is aligned with a predicted site in *D. pseudoobscura.*

o The predicted site overlaps with a predicted site in *D. pseudoobscura.*

n Corresponding 3′UTR sequence in *D. pseudoobscura* is not available. The predicted sites in boldface are covered by the tested RNA probes.

**Figure 3:**
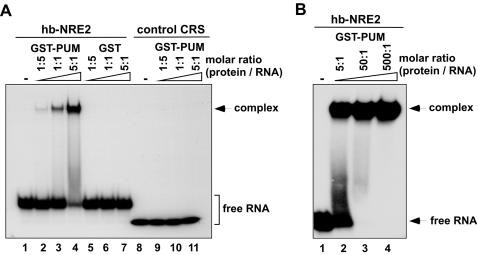
Binding of Pum to *hb* NRE2. (A) EMSA showing the specific binding of recombinant *Drosophila* Pum to radiolabeled *hb* NRE2 RNA. A total of 10 fmol of radiolabeled hb-NRE2 or control CRS RNA was used in each lane. Various amounts of recombinant GST-Pum or GST control proteins were used in different lanes: 2 fmol in lanes 2, 5, and 9; 10 fmol in lanes 3, 6, and 10; 50 fmol in lanes 4, 7, and 11; no protein in lanes 1 and 8. (B) EMSA showing the complex formed between Pum and hb-NRE2 at a higher molar ratio of protein to RNA. A total of 10 fmol of radiolabelled hb-NRE2 RNA was used in each lane. Recombinant GST-Pum proteins used in lanes 1, 2, 3, and 4 were 0 fmol, 50 fmol, 500 fmol, and 5 pmol, respectively.

We next sought to determine the binding specificity of the predicted NRE-like elements. To this end, we carried out electrophoretic mobility shift assay (EMSA) using purified GST-Pum, which bears the RNA-binding domain of *Drosophila* Pum (amino acids 1091–1533) fused with an N-terminal GST tag and has been shown to maintain the full binding activity of the wild-type Pum protein [6],[7]. The second NRE element (NRE2) of *hb* served as a positive control for Pum binding, whereas a random control RNA sequence, CRS that does not resemble an NRE-like element served as a negative control. Under the experimental conditions used, GST-Pum bound to *hb* NRE2 with high affinity, but did not bind to the control RNA sequence CRS, as shown in Figure 3A. In a parallel control experiment in which GST-Pum was substituted by GST alone, no protein–RNA complex between GST and *hb* NRE was formed, ruling out the possibility that the complex between GST-Pum and *hb* NRE was generated by non-specific binding of GST to RNA. We also note that, under our experimental conditions, only one complex was formed between GST-Pum and *hb* NRE as we increased the concentration of GST-Pum, consistent with the presence of a single Pum binding site in a single *hb* NRE (Figure 3B).

**Figure 4:**
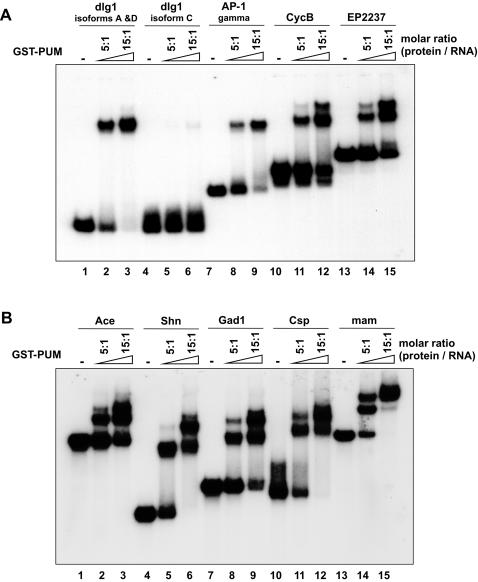
Binding of Pum to the Predicted NRE-Like Elements. (A,B) A total of 10 fmol of radiolabeled RNA from the 3′UTR of the predicted genes was used in each lane. For each RNA sample, recombinant GST-Pum proteins were added in three different amounts: 0 fmol, 50 fmol, and 150 fmol. Electrophoresis was carried out on a 5% native polyacrylamide gel at 150 V at room temperature.

Next, we determined the binding specificity of the predicted NRE-like elements by EMSA. As shown in Figure 4A and 4B, Pum binds, albeit with different affinities, to all these predicted elements, except for *dlg1* isoform C, which was not bound by Pum at all, even at a high molar ratio of protein to RNA. However, *dlg1* transcript isoform C shares a different 3′UTR sequence from *dlg1* transcript isoforms A and D. For the RNAs from *hb* NRE, *dlg1* isoforms A and D, and *AP-1 gamma*, only one complex was formed upon binding of Pum. For the remaining RNAs, two or more complexes were formed, suggesting the existence of more than one Pum-binding site.

To evaluate the relative binding affinities of these NRE-like elements, we quantified the EMSA results on a phosphorimager (Table 1). At the 5∶1 molar ratio of protein to RNA, 67.9% of *hb* NRE was bound by Pum. Under the same experimental condition, greater than 50% of the transcripts from *dlg1* (isoforms A and D), *shn, Csp,* and *mam* were bound by Pum, suggesting that NRE-like elements in these transcripts have strong Pum-binding activities comparable to *hb* NRE. On the other hand, at the same 5∶1 molar ratio of protein to RNA, transcripts from *Ace, AP-1gamma, EP2237*, and *Gad1* were largely unbound by Pum and showed weaker but substantial Pum-binding activities. We also tested Pum-binding activity of a 142-nt RNA fragment consisting of *CycB* TCE and flanking sequences (nts 400–541 of 3′UTR of *CycB* mRNA) and found that *CycB* TCE was able to bind to Pum with a much lower affinity compared to *hb* NRE (Figure 4A, lanes 10–12, and data not shown).

### Model Evaluation with Binding Data

As a validation of our PWM models, we calculated the correlation coefficient between the prediction scores and the binding affinities. The correlation for NRE_M10 is statistically significant (cor = 0.67, *p* = 0.017) whereas the correlation for NRE_M8 is weaker and not statistically significant (see Figure S1 for details). This suggests that NRE_M10 is more accurate than NRE_M8, supporting the assumption behind NRE_M10, i.e., only Box B is important for Pum binding.

### Expression Profiling of Putative Pum Targets During Memory Formation

While the validity of our model is also supported by our in vitro binding experiments, we decided to use an in vivo assay to validate our target prediction method for a few of the putative targets. Because we were interested in targets with potential relevance to behavioral plasticity, we decided first to quantify transcript levels for each candidate in response to behavioral training that induces long-term memory.

Expression profiling after experience-dependent memory formation indicated that the regulatory pathway for local translation (including Pum) is transcriptionally induced by spaced training [12]. Thus we reasoned that mRNA levels of some of Pum targets might also be regulated. Using quantitative (real time) PCR (QPCR), we measured expression levels after spaced versus massed training for each of the putative Pum targets that showed robust binding in vitro. Two of them, *Ace* and *dlg1*, were significantly induced 6 hours after spaced training (fold change = 1.58, *N* = 8, *p* = 0.0036 for *Ace*; fold change = 1.56, *N* = 8, *p* = 0.0068 for *dlg1*). While we do not understand why transcriptional responses for Pum's targets are in the same direction as that of Pum, this may reflect global transcriptional increases versus local translational repression (see Discussion). These two candidate target genes were chosen for in vivo assays.

### In Vivo Confirmation of Predicted NRE Elements

To validate our target prediction method in vivo, we chose to use a Pum response assay described previously [1]. This assay relies upon the requirement that maternally supplied *hb* mRNA be repressed by Pum/Nos in posterior regions of the early embryo. We started with a canonical genomic *hb* rescuing transgene in which the endogenous NRE motifs were deleted. In the absence of functional NRE elements, this construct causes a dominant sterility in transgenic females due to ectopic *hb* translation in the posterior half of the embryos produced. Such embryos are unable to form abdominal segments. Insertion of a functional NRE motif into this canonical construct restores Pum-mediated repression in the posterior, allowing production of viable progeny. Using this strategy, we tested the functional capacity of the predicted NRE motifs from *Ace* and *dlg1*. We chose these two putative targets because they showed relatively strong in vitro binding and also because both transcripts are induced by spaced training.

**Figure 5:**
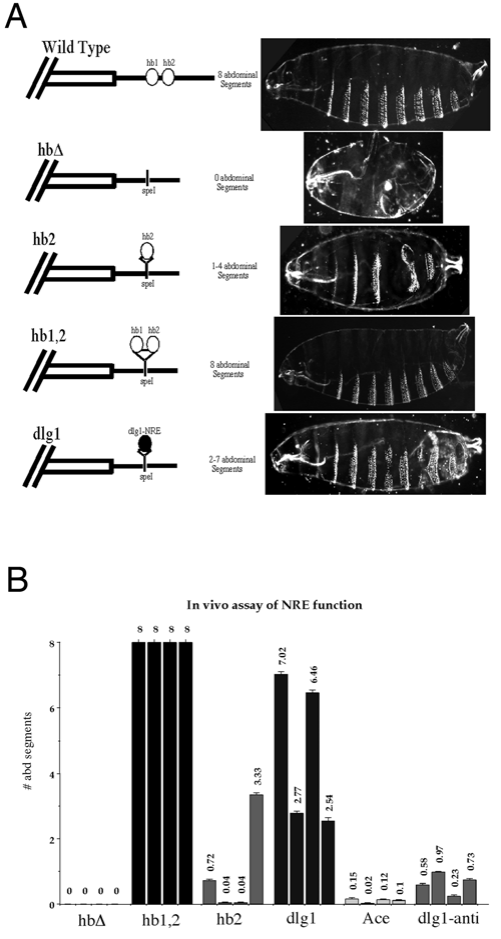
In Vivo Confirmation of Predicted NRE Elements. (A) Cartoon representations of the *hb*-transgene constructs and representative examples from cuticle preparations of corresponding transformant lines, showing normal/abnormal or rescued/partial rescued abdominal segmentation. Transgenic lines containing both *hb* NREs either in the normal context (wild type) or reinserted into the deletion construct (hb1,2) are fully regulated by Pum and yield embryos with the normal complement of 8 abdominal segments. Deletion of both of these NREs (hbΔ) prevents Pum-dependent translational repression leading to complete absence of abdominal segmentation. Insertion of either one *hb* NRE (hb2) or the predicted NRE from *dlg1* (dlg1) are each sufficient to partially restore abdominal development. (B) The average numbers of abdominal segments are shown for each of four transformant lines resulting from each of the *hb*-transgene constructs. A total of 72–140 embryos of each transformant line were analyzed to count the number of abdominal segments to generate the mean number. (See Table S4 for frequency distribution of each line).

**Figure 6:**
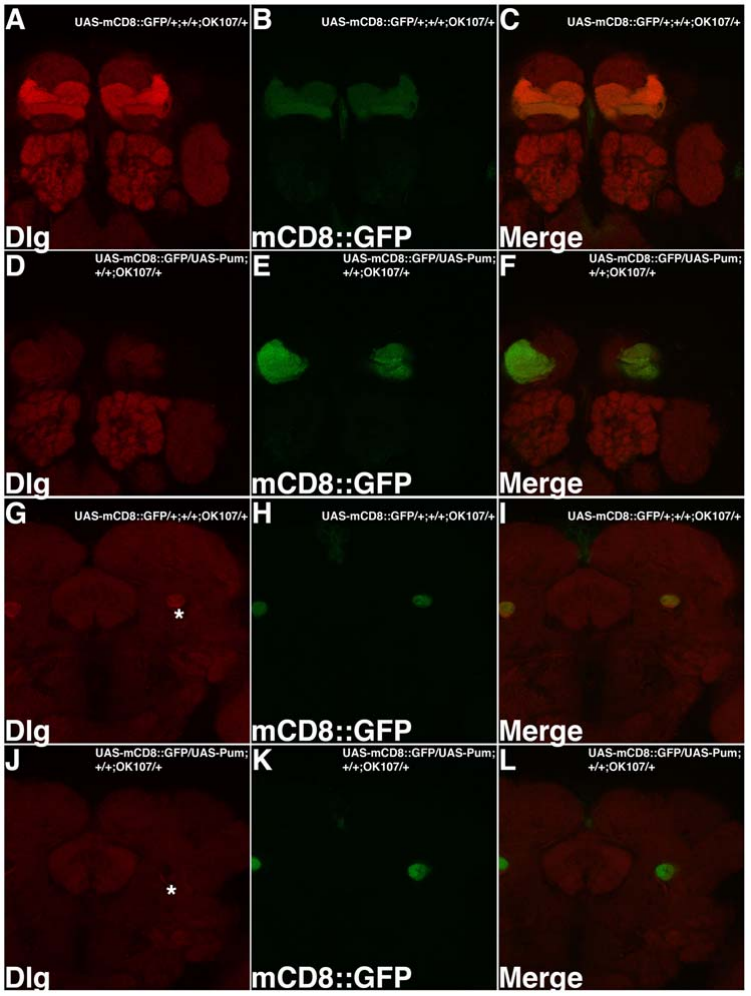
Dlg Is Repressed by Over-Expression of Pum in MB Kenyon Cells. MB expressing Gal4 line (OK107) was used to drive expression of both UAS-mCD8::GFP (green) and UAS-Pum transgenes. Optical sections of the MB lobes (A–F) or MB peduncle (G–L) are shown. In wild type (A–C, G–I), Dlg expression (red) is detected both in MB lobes and AL (A) as well as in the MB peduncle (G). In contrast, Dlg expression is dramatically reduced in the MB lobes (D) and peduncle (J) of Pum over-expressing MBs. AL glomeruli, also stained by Dlg antibody, serve as an internal control. We also noticed that when Pumilio is over-expressed in MB, there is MB developmental defect in MB lobe structure (visualized by GFP and shown in [E]). Despite this, GFP levels appear normal in the peduncle (K). Asterisk indicates peduncles where Dlg expression can be detected in wild type MB but is dramatically reduced in Pum over-expressing MB. As an additional confirmation, we used a series of Gal4 drivers that express preferentially in subsets of MB neurons (data not shown). Among all these Gal4 drivers, OK107 produced the strongest downregulation of Dlg as stated above. 238Y and c739 produced the second strongest effects: Dlg expression level is significantly reduced in the peduncle of the UAS-Pum; 238Y brains and is dramatically reduced in the α/β lobes of the UAS-Pum; c739 brains. We did not observe MB structural defects with either of these lines We also did not observe effects with Pum over-expression using c747 or c309. This likely is due either to differences in expression levels, timing or neuron number. Pum over-expression crosses using elav, MJ85b, 247 and 201Y and GH146 Gal4 lines were lethal during development.

We generated a series of *hb*-transgene constructs (Figure 5 and Table S4) in which the two endogenous *hb* NREs had either been deleted entirely (**hbΔ**), replaced with a single *hb* NRE, NRE2 (**hb2**), had both *hb* NRE elements re-inserted (**hb1,2**), replaced with putative NRE elements from *Ace* or *dlg1* genes (**Ace** or **dlg1**), or replaced with an anti-sense version of the predicted *dlg1* NRE (**dlg1-anti**). We found that the predicted NRE from *dlg1* is sufficient to partially restore abdominal patterning when compared with **hb1,2** (Figure 5A and 5B), which provided full rescue as in Wharton and Struhl [1]. It is worth noting that the rescue observed with the single *dlg1* NRE is superior to that observed with a single copy of the *hb* NRE (Figure 5A and 5B). Consistent with a previous observation by Wharton and Struhl [1], a single *hb* NRE (**hb2**) yields partial rescue. In contrast, control transgenic lines in which no functional NRE was provided, or in which the *dlg1* NRE was inserted in opposite orientation (**dlg1-anti**) generate progeny nearly devoid of abdominal segments (Figures 5A and 6B, and Table S4). It is also worth mentioning that we failed to observe a rescue of normal abdominal segmentation when using another *hb*-transgene construct (**dlg1-full**), in which the two endogenous *hb* NREs are replaced by a longer version of the transcript, a 1.2-kb sequence including the predicted NRE element from the 2.8-kb sequence of *dlg1* 3′UTR (Table S4). The lack of rescue with this construct may be caused by the artificial context of the transcript resulting from insertion of such a large heterologous fragment into the *hb* 3′UTR. We also failed to observe any rescue when using a *hb*-transgene construct in which *hb* NREs were replaced with putative NRE elements from *Ace,* indicating *Ace* might not function as a Pum target in an in vivo context despite positive results in computational search and biochemical validation.

### In Vivo Confirmation of *dlg1* as a Target of Pumilio in a Neuronal Context

The above data support the conclusion that the *dlg1* mRNA contains a Pum binding site that can confer translational repression to a heterologous reporter system in the embryo. We next sought to test whether the endogenous *dlg1* mRNA can be regulated by Pumilio in a relevant neuronal context. Because of our interest in olfactory memory, we chose to test for Pum-mediated regulation of Dlg in the adult mushroom body (MB), which is an anatomical site of memory storage [23]–[25]. We first used a monoclonal antibody against Dlg to examine the distribution of Dlg protein in brains of wild-type animals. Consistent with Ruiz-Canada et al. [26], we found that Dlg is widely distributed in the adult brain, with elevated levels in antenna lobes (AL) and mushroom bodies (Figure 6A). We then tested whether transgenic over-expression of Pum in MB was sufficient to reduce the endogenous Dlg expression. To do this, we used a MB Gal4 enhancer trap line OK107 [27] to drive the expression of both UAS-mCD8::GFP and UAS-Pumilio transgenes in the same brain. The GFP expression permitted independent visualization of the MB neuronal architecture and also served as an internal control for the distribution of Dlg.

Our imaging studies support two conclusions. First, we found that transgenic expression of Pum in MB Kenyon cells results in a dramatic reduction of Dlg expression levels. Importantly Dlg expression in AL appears unaffected (Figure 6A). In addition, the GFP expression in MB neurons appears at normal levels. This observation strongly supports the hypothesis that endogenous Dlg expression can be repressed by Pum in the CNS. Second, we also noticed that transgenic over-expression of Pum causes a severe defect in the elaboration of the axonal projections of MB neurons. This is evident in the expression of UAS-mCD8::GFP, which permits visualization of the entire MB neuronal architecture. In wild type animals, MB Kenyon cell axons dive ventrally and anteriorly along the peduncle. They then bifurcate into distinct vertical (α and α′) and horizontal (β, β′ and γ) lobes, which contain the axon terminals. In contrast, the MBs of Pum over-expressing animals do not form normal lobe structures. Instead, the axons appear to prematurely terminate just medial to the peduncle.

**Figure 7:**
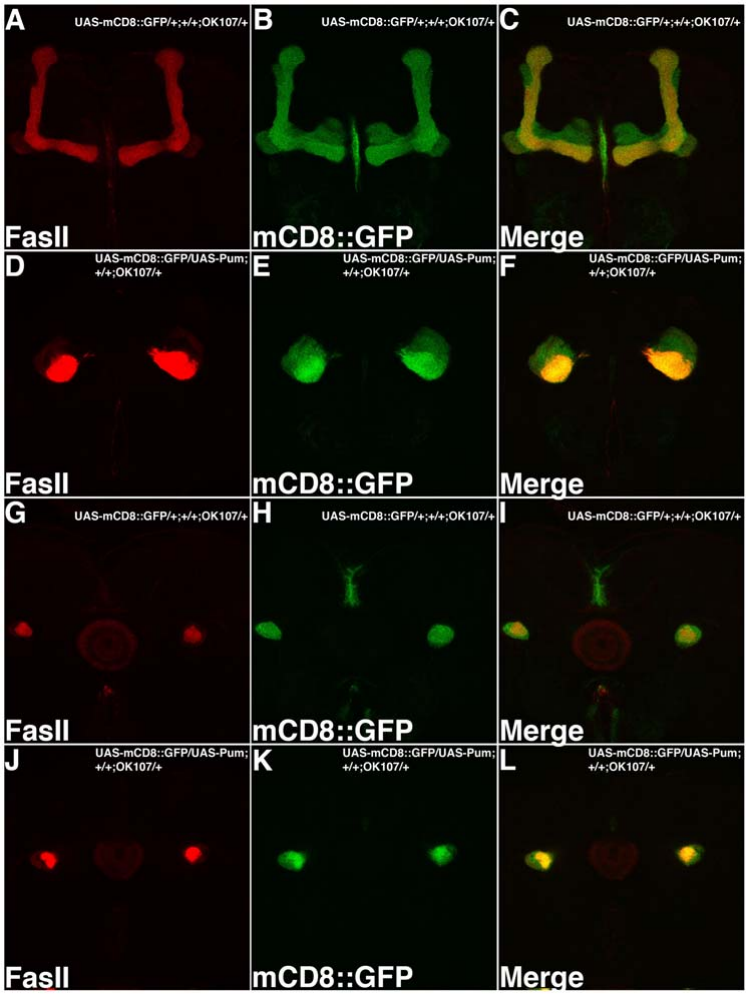
FasII Levels Appear Unaffected by Pum Over-Expression. MB expressing Gal4 line (OK107) was used to drive expression of both UAS-mCD8::GFP (green) and UAS-Pum transgenes. Optical sections of the MB lobes (A–F) or MB peduncle (G–L) are shown. In both wild type (A–C and G–I) and Pum over-expressing MBs (D–F and J–L), FasII expression is detected both in MB lobes (α/β [A] and [D]) as well as in the MB peduncle ([G] and [J]). The MB developmental defect in Pum over-expressing MBs can be visualized by both GFP (E) and FasII staining (D).

The above observation suggests the interesting possibility that Pum normally plays a key developmental role in elaboration of MB structure. While we cannot rule out neo-morphic effects of Pum over-expression, these findings nevertheless are consistent with the previous observations of Pum's role in dendrite morphology [10]. At the same time, however, we were concerned that the decreased accumulation of Dlg protein that we observed with Pum over-expression could be an indirect consequence of the MB structural defects. We used several strategies to rule this out. First, we made careful observation of Dlg expression levels in the peduncle in both wild type control and Pum over-expressing brains (Figure 6A). Unlike the lobes, which are largely absent from these animals, the peduncle is intact. GFP expression in the peduncle was used as a reference. Second, we used a monoclonal antibody against FasII, which like Dlg, is expressed at elevated levels in MB Kenyon cell neurons (although mostly α/β). This permitted a second independent means to image the MB of the same animals and also provided expression of a second endogenous protein as a control. Both of these experiments support the conclusion that Dlg expression per se is reduced in Pum over-expressing animals because both GFP and FasII protein levels are un-altered in the residual lobes and in the peduncle (Figure 6B and Figure 7). Finally, we used several additional MB expressing Gal4 drivers to confirm the key observation that ectopic Pum can down regulate Dlg (see Figure 6 legend; data not shown). The magnitude of the effects on Dlg expression varied depending on expression levels, timing and number/type of MB neurons labeled. Nevertheless, we observed decreased Dlg immuno-labeling both with MB Gal4 line C739 and 238Y (Figure 6 legend and data not shown).

## Discussion

The bioinformatic prediction of mRNA targets for sequence-specific RNA binding proteins continues to be a significant challenge. In most cases, biologically relevant motifs are hard to define, in part due to the unknown impact of secondary structure. This is confounded by the fact that in vivo assays to validate predictions are often not trivial. One approach to identify targets is to use genome-wide detection of mRNAs that directly associate with an RNA-binding protein. This approach was used with success [15] to identify putative Pum-associated mRNAs from ovaries and early embryos. In this study, we have taken a different approach to identify neuronal targets that might underlie Pum's role in memory. We took advantage of: (1) the availability of well characterized structural and functional information about Pum-HD:RNA interactions; (2) several conserved NRE elements that had been described for the *hb* and *bcd* genes; (3) the availability of a robust in vivo functional assay [1], and (4) in vivo imaging of one target gene's expression to validate our predictions. We have identified a group of putative neuronal targets of Pum, including *dlg1* and *Ace*, both of which are also induced during memory consolidation. In the case of *dlg1*, the identified NRE appears capable of functioning both in a heterologous in vivo context of the early embryo and an endogenous one in the adult brain (Figures 5 and 6).

Our results also suggest that the binding specificity of Pum is conserved between *Drosophila* and mammals, as previously noted in Wang et al. [17], which is consistent with the observations that human Pum2 binds to the *Drosophila* NRE sequence [28],[29]. First, NRE_M10, which is based on assumptions derived from the human Pum-RNA crystal structure, performed best among the three motif models constructed with known Pum targets in flies. Second, a motif derived from mouse PUM2 SELEX data, MmSelex_M8 (“Conservation of Pum binding specificity between fly and mouse” in Text S1 and Figure S2), fit well with the *Drosophila* Pum binding data from EMSA. Furthermore, this conservation of Pum binding specificity may be extended to non-mammalian vertebrates, as *Xenopus* Pum has been shown to bind *Drosophila hb* NRE [18],[30]. In fact, the RNA-binding domain of *Drosophila* Pum is very similar to that in human, mouse and *Xenopus* (amino acid identity ≥78%).

The fact that prediction scores of NRE_M10 and MmSelex_M8 are well correlated with in vitro binding data demonstrates the validity of these two models for Pum binding site prediction. The predicted hits by these two models in the synaptic gene set are significantly higher than random (Figure S3), further demonstrating their validity and also suggesting that a number of synaptic genes are likely regulated by Pum. In the case of *dlg1*, our in vivo evidence indicates that the predicted NRE can function, not only in context of the *hb* 3′UTR, but also in CNS while Pum is over-expressed.

Comparing our synaptic gene set with the pulled-down targets from Gerber et al. [15], 27 (18%) genes are in the adult specific target list. Only one gene overlaps with the embryo specific targets, presumably because the embryo specific target list is much smaller. Our predicted Pum targets using NRE_M10 and mmSelex_M8 are significantly enriched with experimentally pulled-down targets (36% and 30%, respectively, see Figure S5 for more details). Although our NRE models, NRE_M10 and mmSelex_M8 were constructed from a very limited number of training sequences, the motif patterns match closely with the consensus Pum binding site published in Gerber et al. [31], especially in the 8-nt core motif. These all validate the effectiveness of our method. Of course, further improvement can be made with more high confidence training sequences.

Studies in diverse organisms strongly indicate that sequences around BoxB play a major role in binding to Puf proteins [15],[17],[18],[31] although BoxA may affect the binding affinity to some extent [32]. Interestingly, the binding specificities appear to vary among Puf family members even though their RNA-binding domains are highly conserved. For example, Puf3, Puf4 and Puf5 in yeast appear to recognize similar motifs but in different lengths [31]. A recent finding by Opperman et al. [33] shed a light on this. It is indicated that small structural difference in the RNA-binding domain may require extra spacer nucleotides in the binding site. This BoxB related motif, hallmarked with UGUA tetranucleotide, may represent the most prevalent binding sites for Pum or even Puf family proteins. However, other types of binding sites may also exist as we will discuss below.

Notably, Pum binds to a 142-nt RNA harboring *CycB* TCE with a lower affinity than *hb* NRE under our experimental conditions. *CycB* TCE was initially proposed due to its resemblance to *bcd* and *hb* NRE, and was required for translational repression control [22]. This cis-acting element was able to bind GST-Pum [5],[34], but not the purified Pum RBD or native embryonic extracts [5],[34]. Indeed, *CycB* TCE has a lower score according to our matrix. A new element downstream of TCE has recently been proposed and been shown to bind to Pum in gel mobility shift experiments and, when substituted for the native *hb* NRE in a chimeric *hb* mRNA, was able to mediate *CycB*-like regulation on *hb* mRNA [5],[34]. Intriguingly, our matrix also predicts a Pum-binding site with high score (ATTGTGCAAA, nts 561–570 of 3′UTR of *CycB* mRNA) in the RNA fragment used in these experiments. Our predicted site is close to the NRE element proposed by Kadyrova and colleagues, but not the same. Further work needs to be done to address this discrepancy. It is also worth mentioning that there are several significant differences between regulation of *CycB* mRNA and *hb*/*bcd* mRNAs [5],[34]. In contrast with *bcd* and *hb*, for example, regulation of *CycB* is Brat-independent. Kadyrova et al. [5] have demonstrated that in the case of *CycB*, Pum binding seems important only to recruit Nanos, because artificially tethering Nanos to the 3′UTR bypasses the requirement for Pum binding. This is in contrast to Pum's regulation of *hb*. Thus it seems that there are significant differences between the Pum-binding sites in *CycB* mRNA and those in *hb* and *bcd* mRNAs, as proposed previously [8]. Related to that, in the minimal 51 nt eIF-4E 3′UTR sequence bound by Pum [8], only one binding site is predicted by NRE_M8 with a score just above the cutoff value 7.5, suggesting the Pum binding to eIF-4E 3′UTR may be also different from *hb* and *bcd*. Discovery of additional Pum targets from a variety of cell types and biological contexts may uncover the relationship between NRE sequence and regulatory mechanism.

To our knowledge, this is the first study to characterize and predict Pum-binding sites with a PWM approach, which is typically more sensitive and more precise than consensus methods [21]. Our in vitro binding assay of Pum on a subset of the predicted targets provides a measure of validation of our motif models. Like Pum, two of these targets, *Ace* and *dlg1*, also appear to be transcriptionally induced after spaced training relative to massed training, suggesting that these are relevant targets for memory formation. We do not know why both a translational repressor and its putative targets are transcriptionally induced. It may be that transcripts are increased on a cell-wide level, while translation is spatially regulated within neurons. In the case of *dlg1*, our in vivo evidence supports the conclusion that the predicted NRE can mediate Pum-dependent repression both when it is in the context of the *hb* 3′UTR and in the endogenous *dlg1* transcript in the CNS. Thus, our findings directly predict that *dlg1* is a synaptic target of Pum.

Dlg is the sole *Drosophila* member of a family of membrane-associated guanylate kinases (MAGUKs) that in mammals have been shown to play a key role in assembling the post-synaptic density in glutamatergic synapses. In *Drosophila*, Dlg expression is both pre- and post-synaptic at Type I boutons at the NMJ, and mutants exhibit post-synaptic structural defects as well as increased transmitter release [35],[36]. Dlg is thought to play a key role in clustering GluRIIB receptors at the NMJ [37] as well as Shaker K^+^ channels throughout the CNS [26].

Like Dlg, Pum also appears to have both pre- and post-synaptic effects at the NMJ and is co-localized with Dlg at Type I boutons [8]. In addition to morphological effects on synapse structure, Pum appears to regulate excitability via an effect on expression of *para* Na^+^ channels [9],[11],[38]. The regulation of *para* may be direct, or may depend upon Pum's putative role in regulating translation of eIF-4E [8]. Pum expression itself is activity-induced and is induced by behavioral training that results in long-term memory [9],[12]. Thus, one reasonable hypothesis is that activity-dependent increases in Pum expression play a homeostatic role by reducing excitability via repression of *para* [38]. *para* is in our list of synaptic genes, yet our models did not predict any Pum binding sites in its 3′UTR. That is not surprising since Mee et al. [9] reported NRE-like sequence located in its 5′UTR. Therefore, a different mechanism may be involved in the regulation of *para* by Pum.

Our findings suggest that an additional role of Pum is direct regulation of *dlg1* expression, thereby antagonizing the effects of Dlg on neuronal structure and/or function. We do not yet know whether other classic factors (Nanos and Brat) that cooperate with Pum in early embryos are also required in the translational control of Dlg in neurons. Further investigation also will be required to separate the roles of Pum in neuronal development and memory formation. Ultimate confirmation that Pum-dependent repression of *dlg1* and the other predicted NRE-containing genes underlies Pum's role in neuronal structure, function and memory will also require additional examination.

## Materials and Methods

### Synaptic Gene Collection

Synaptic genes were collected based on GeneOntology (GO) terms in the Berkeley *Drosophila* Genome Project (BDGP, http://www.fruitfly.org/) Release 3.1 annotation and keyword search in the FlyBase Vocabulary Report (http://flybase.org/) of gene expression. GO terms involved in neurotransmitter metabolism were not considered to relate directly to synaptic functions, and were thus excluded. 68 genes were obtained from the GO annotation and 132 genes were obtained from FlyBase search using the keyword “synapse.” Among those, a total of 151 genes were mapped to Release 3.1 *Drosophila* genome (with CG ID) and were used for further analysis (Table S5).

### 3′UTR Sequence Collection

Sequences of mRNA or genomic DNA that contain complete 3′UTRs of *hb* and *bcd* from different fly species were retrieved from GenBank. The GenBank accessions are listed in Figure 1B.

The 3′UTR sequences of all annotated genes for *D. melanogaster* were retrieved from BDGP Release 3.1 annotation. Putative *D. pseudoobscura* 3′UTR sequences were obtained based on whole-genome alignment between *D. melanogaster* and *D. pseudoobscura* produced by the BDGP at Lawrence Berkeley National Laboratory (http://pipeline.lbl.gov/). Distinct 3′UTR sequences of the mapped 151 synaptic genes are included in Tables S1, S2, S3, S4, S5.

### Construction of Fly NRE Matrix Models

The Gibbs Sampler program ([39]; also refer to http://bayesweb.wadsworth.org/gibbs/gibbs.html) obtained from C. E. Lawrence's group was used to perform local multiple sequence alignment to identify the motif model. The base-frequency matrix output from the program was converted into the log-odds PWM with a background nucleotide frequency derived from all 3′UTRs in the genome of *D. melanogaster*, i.e.,
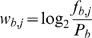
where *w_b,j_* is the matrix weight for base *b* at position *j*, *f_b,j_* is the frequency of base *b* at position *j* and *p_b_* is the background frequency of base *b*. *b* = A, C, G or T, *j* = 1 … *n* for a PWM of length *n*.

Input sequences to Gibbs Sampler included known NREs in *hb* and *bcd* of *D. melanogaster* and their corresponding sequence segments in other fly species (Figure 1B, in DNA letters without gaps). 3′UTR sequences for *CycB* (*melanogaster* and *pseudoobscura*) and *eIF-4E* (*melanogaster* only) were also included.

### Search for New Pum Targets

Pattern search was implemented with a Perl script as a regular expression match. Weight matrix scan on sequences was performed with an R script. For a PWM of length *n*, the score of a target sequence segment *t = b_1_b_2_ … b_n_*, is:
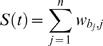
where *j* is the position in the PWM, *b_j_* is the *j* ^th^ base of the target sequence.

We searched in the 3′UTR sequences of all 151 synaptic genes, including their distinct splicing variants in *D. melanogaster*. The matrix score cutoff was selected so that most of the known NREs scored above the threshold. Corresponding putative 3′UTR sequences of *D. pseudoobscura* were also searched when available. We define a predicted site in *D. melanogaster* as conserved if this site is aligned or overlaps with a predicted site in *D. pseudoobscura* in the LAGAN alignment provided by BDGP.

### Purification of GST-PUM

The plasmid R6646 that encodes amino acids 1091–1533 of *Drosoplila* Pum as a fusion with GST [7] was a gift from Dr. Robin Wharton. The protein was expressed in *E. coli* and purified by affinity chromatography on glutathione-Sepharose (Amersham Biosciences) by standard procedures.

### In Vitro Transcription

Transcription templates for the predicted NRE-like elements were obtained by PCR from *D. melanogaster* genomic DNA. A T7 promoter was added at the 5′ terminus of the template by PCR. PCR products were purified and used as templates for in vitro transcription, which was done as described [40]. RNA transcripts were purified by electrophoresis on an 8% or 4.5% polyacrylamide/7M urea gel.

### EMSA

EMSA was done as described [6]. The *hunchback* NRE2 sequence used was AUUAUUUUGUUGUCGAAAAUUGUACAUAAGCC. The random control RNA sequence (CRS) is GGUAGUGCAUACAACUUCCUU. Binding reactions were carried out by mixing 10 fmol radiolabeled RNA with variable amounts of purified GST-Pum in a 10 μl binding buffer containing 0.1 mg/ml BSA, 10 mM Hepes/KOH pH 7.4, 50 mM KCl, 3 mM MgCl_2_, 1 mM EDTA, 0.1 mg/ml yeast tRNA, 2 mM dithiothreitol, 0.01% (w/v) Tween-20, 0.2 U rRNAsin (Promega), and 10% (v/v) glycerol. The protein-RNA complexes were allowed to form for 20 min at room temperature, followed by electrophoresis on a 5% non-denaturing acrylamide gel in 1× TBE buffer. The gel was dried, followed by autoradiography at −70°C or quantification on a phosphorimager (Fuji).

### Quantitative PCR

RNA isolations were performed with Trizol (Invitrogen) as described before [12], with the following modifications. After the Trizol step, samples were treated with DNase I (Promega, 5 U) for 30 min (37°C) and then were extracted with phenol/chloroform/iso-amyl alcohol (Invitrogen), precipitated with ethanol, and resuspended in DEPC-treated water. RNA quality was tested using an Agilent Bioanalyzer 2100 and RNA 6000 Nano Chips. Reverse transcription reactions were performed with 5.0 µg RNA per reaction with an oligo dT primer and Taqman reverse transcription reagents (Applied Biosystems) in 100 µl total volume. PCR quantification was performed by using 4 µl of the above RT product per reaction on a real-time PCR machine (7900 HT, Applied Biosystems) using Taqman probe and Taqman reagents (Applied Biosystems) according to the manufacturer's protocols. Gene-specific primers and Taqman probes had the following sequences:

Ace: primers: 5′-GCACTACCCAAGACAAATTTTATCGAAA-3′ and 5′-GCCCCGTACTACGCTTACAA-3′; probe: 5′-CACATTTTCGATCGATTCTT-3′

dlg1: primers: 5′-ATCCGCATAATAATGTAAACTACGACAGAA-3′ and 5′-ACTCATTATATAGGTTTAAATCAACGCGAfCAA-3′; probe: 5′-CAAATTCAATTTCTCCTTTTTTCC-3′

TBP: primers: 5′-GCATCATCCAAAAGCTCGGTTT-3′ and 5′-GAGCCGACCATGTTTTGAATCTTAA-3′; probe: 5′-CCCTGCAAAGTTCC-3′

Prior to QPCR quantification of Pum targets, all primers and probes underwent the linearity test using 1, 2 and 4 µg RNA for RT reaction. Expression levels were normalized to Drosophila TBP transcript levels. TBP was confirmed as an unchanged control by comparing in excess of 100 RNA extractions each after spaced and massed training (data not shown). All reactions were done in parallel by using at least eight independent RNA isolations for each group, with each RNA isolate being assayed once. Normalized threshold values (Ct) were subjected to parametric t-tests, with significance levels set at alpha = 0.05.

### In Vivo Assay for NRE Function

The in vivo function of the predicted NRE-like elements was tested as described by Wharton and Struhl [1]. Briefly, the selected NRE-like elements and control DNA were each cloned and inserted into the SpeI site of plasmid p1809, which bears a *hunchback* genomic rescuing construct with a deletion of NRE elements. An Asp718I-BamHI fragment containing each modified *hunchback* gene was cut out from the resulting plasmid and inserted into the P-element transformation vector CaSpeR4 digested with the same restriction enzymes. The resulting constructs were injected separately into w^1118^(isoCJ1) [12] recipient embryos and transformant lines were isolated by standard procedures via the BestGene, Inc.. In all cases, only male progeny were bred to avoid selecting non-expressing inserts. For each modified *hunchback* gene, four independent transformant lines were analyzed for the effects on segmentation pattern in embryos. NRE function in each line was tested by collecting embryos from heterozygous females. Cuticle preparations were analyzed according to Wharton and Struhl [1].

### Flystocks and Crosses

A stock (5137; OK107) which is homozygous for both MB-specific Gal4 driver OK107 (on chromosome IV) and UAS-mCD8::GFP (on chromosome II) was crossed with wild type w^1118^(isoCJ1) or UAS-Pumilio (on chromosome II) homozygotes flies.

### Immunohistochemistry and Image Acquisition and Processing

Adult brains were dissected in 1× PBS, fixed in 1× PBS containing 4% formaldehyde for 30 minutes, and blocked in penetration/blocking buffer consisting of 1× PBS, 2% Triton and 10% normal goat serum (Jackson ImmunoResearch Laboratories, Cat. 005-000-121) for 2 hours at 4°C. Then dissected brains were placed in primary antibody (1:20 dilution in Dilution Buffer containing 0.25% Triton and 1% normal goat serum in 1× PBS) for overnight at 4°C. After washing by Washing Buffer (1% Triton, 3% NaCl in 1× PBS) for 4×10 minutes in room temperature, dissected brains were placed in secondary antibody (1:200 dilution in Dilution Buffer) for overnight at 4°C. The following antibodies were used: monoclonal anti-discs large-s antibody 4F3 (Developmental Studies Hybridoma Bank at the University of Iowa) as primary antibody for Dlg staining, monoclonal anti-Fasciclin II-s antibody 1D4 (Developmental Studies Hybridoma Bank at the University of Iowa) as primary antibody for FasII staining, Cy3 conjugated AffniPure Goat Anti-Mouse IgG (H+L) (Jackson ImmunoResearch Laboratories, Cat. 115-165-003) as secondary antibody. Finally, the brains were washed by washing buffer for 4×10 minutes at room temperature, treated with FocusClear (CelExplorer Labs, Cat. FC-101) for 10 minutes and mounted onto slides with MountClear (CelExplorer Labs, Cat. MC-301).

Confocal stacks of brains were acquired using a ZEISS LSM 510 confocal microscope. Following confocal settings were used: 40× water immersion lens, 1 µm spacing in the *z*-axis and 1024×1024 resolution in *x*- and *y*-axes. The Cy3 signal is captured by HeNe1 543nm laser and GFP signal is captured by Argon/2 488nm laser. All brains were scanned from the anterior to the posterior to ensure good resolution of MB. The raw data were processed by LSM Image Browser Rel.4.2 (ZEISS) and further arranged into figures by Adobe Photoshop CS2.

## Supporting Information

Text S1 Supplementary Material
(0.05 MB DOC)

Figure S1 Correlation Between Matrix Prediction Scores and Pum-Binding Affinities. The abscissa is the measured percentage binding of Pum to the mRNA target. The ordinate is the prediction score, which is the maximum matrix score of all the sites in a sequence. The 12 data points represent 12 mRNA sequences (nine test sequences in Table 1 and three control sequences). The Pearson correlation coefficient (cor) and its p-value are shown in the upper left corner. (A) Correlation for matrix NRE_M8. (B) Correlation for matrix NRE_M10.
(0.16 MB TIF)

Figure S2 MmSelex_M8 Matrix and the Correlation of Its Prediction Scores to Pum-Binding Affinities. (A) Base-frequency matrices obtained using Gibbs Sampler with mouse SELEX sequence data from White et al. [41]. Position 5 is a motif gap as in Gibbs output, which means that the base in this position is irrelevant. DNA notation is used as in Figure 2. (B) Correlation between matrix prediction scores and Pum-binding affinities for MmSelex_M8. Notations are the same as in Figure S1.
(0.19 MB TIF)

Figure S3 Estimation of False Positives with Random Shuffle Tests on the 151 Synaptic Genes. Shuffling times n = 500. (A) Matrix NRE_M10. (B) Matrix MmSelex_M8. The gray bars represent the hits with the original matrix. The black bars represent the average hits with randomly shuffled matrices. The error bar is the standard deviation across the 500 shuffling tests.
(0.21 MB TIF)

Figure S4 dlg1 Gene Structure as Shown in the FlyBase Genome Browser. Transcript dlg1-RA and dlg1-RC are located on non-overlapping regions on the fly genome.
(0.06 MB TIF)

Figure S5 Comparison of the Overlap of Our Pum Target Predictions with the Adult Specific Targets from Gerber et al. [15] in the Synaptic Gene Set. Pred+ and Pred− represent the number of our positive or negative prediction, respectively. PD+ and PD− represent the number of positive or negative pulled-down targets from Gerber et al. (2006), respectively.
(0.09 MB TIF)

Table S1 NRE_PAT Predictions
(0.02 MB XLS)

Table S2 NRE_M8 Predictions
(0.03 MB XLS)

Table S3 NRE_M10 Predictions
(0.02 MB XLS)

Table S4 Segmentation Pattern in Embryos of Modified Hunchback Gene Transformant Lines
(0.02 MB XLS)

Table S5 Synaptic Gene List
(0.04 MB XLS)

## References

[1] Wharton RP, Struhl G (1991). RNA regulatory elements mediate control of *Drosophila* body pattern by the posterior morphogen nanos.. Cell.

[2] Murata Y, Wharton RP (1995). Binding of pumilio to maternal hunchback mRNA is required for posterior patterning in *Drosophila* embryos.. Cell.

[3] Gamberi C, Peterson DS, He L, Gottlieb E (2002). An anterior function for the *Drosophila* posterior determinant Pumilio.. Development.

[4] Asaoka-Taguchi M, Yamada M, Nakamura A, Hanyu K, Kobayashi S (1999). Maternal Pumilio acts together with Nanos in germline development in *Drosophila* embryos.. Nat Cell Biol.

[5] Kadyrova LY, Habara Y, Lee TH, Wharton RP (2007). Translational control of maternal Cyclin B mRNA by Nanos in the *Drosophila* germline.. Development.

[6] Zamore PD, Williamson JR, Lehmann R (1997). The Pumilio protein binds RNA through a conserved domain that defines a new class of RNA-binding proteins.. RNA.

[7] Wharton RP, Sonoda J, Lee T, Patterson M, Murata Y (1998). The Pumilio RNA-binding domain is also a translational regulator.. Mol Cell.

[8] Menon KP, Sanyal S, Habara Y, Sanchez R, Wharton RP (2004). The translational repressor Pumilio regulates presynaptic morphology and controls postsynaptic accumulation of translation factor eIF-4E.. Neuron.

[9] Mee CJ, Pym EC, Moffat KG, Baines RA (2004). Regulation of neuronal excitability through pumilio-dependent control of a sodium channel gene.. J Neurosci.

[10] Ye B, Petritsch C, Clark IE, Gavis ER, Jan LY (2004). Nanos and Pumilio are essential for dendrite morphogenesis in *Drosophila* peripheral neurons.. Curr Biol.

[11] Schweers BA, Walters KJ, Stern M (2002). The *Drosophila melanogaster* translational repressor pumilio regulates neuronal excitability.. Genetics.

[12] Dubnau J, Chiang AS, Grady L, Barditch J, Gossweiler S (2003). The staufen/pumilio pathway is involved in *Drosophila* long-term memory.. Curr Biol.

[13] Steward O, Schuman EM (2003). Compartmentalized synthesis and degradation of proteins in neurons.. Neuron.

[14] Vessey JP, Vaccani A, Xie Y, Dahm R, Karra D (2006). Dendritic localization of the translational repressor Pumilio 2 and its contribution to dendritic stress granules.. J Neurosci.

[15] Gerber AP, Luschnig S, Krasnow MA, Brown PO, Herschlag D (2006). Genome-wide identification of mRNAs associated with the translational regulator PUMILIO in *Drosophila melanogaster.*. Proc Natl Acad Sci U S A.

[16] Sonoda J, Wharton RP (1999). Recruitment of Nanos to hunchback mRNA by Pumilio.. Genes Dev.

[17] Wang X, McLachlan J, Zamore PD, Hall TM (2002). Modular recognition of RNA by a human pumilio-homology domain.. Cell.

[18] Bernstein D, Hook B, Hajarnavis A, Opperman L, Wickens M (2005). Binding specificity and mRNA targets of a *C. elegans* PUF protein, FBF-1.. Rna.

[19] MacDonald PM (1990). bicoid mRNA localization signal: Phylogenetic conservation of function and RNA secondary structure.. Development.

[20] Stormo GD, Fields DS (1998). Specificity, free energy and information content in protein-DNA interactions.. Trends Biochem Sci.

[21] Stormo GD (2000). DNA binding sites: Representation and discovery.. Bioinformatics.

[22] Dalby B, Glover DM (1993). Discrete sequence elements control posterior pole accumulation and translational repression of maternal cyclin B RNA in *Drosophila.*. EMBO J.

[23] Heisenberg M (2003). Mushroom body memoir: From maps to models.. Nat Rev Neurosci.

[24] Waddell S, Quinn WG (2001). What can we teach *Drosophila*? What can they teach us?. Trends Genet.

[25] Margulies C, Tully T, Dubnau J (2005). Deconstructing memory in *Drosophila.*. Curr Biol.

[26] Ruiz-Canada C, Koh YH, Budnik V, Tejedor FJ (2002). DLG differentially localizes Shaker K+-channels in the central nervous system and retina of *Drosophila.*. J Neurochem.

[27] Connolly JB, Roberts IJ, Armstrong JD, Kaiser K, Forte M (1996). Associative learning disrupted by impaired Gs signaling in *Drosophila* mushroom bodies.. Science.

[28] Moore FL, Jaruzelska J, Fox MS, Urano J, Firpo MT (2003). Human Pumilio-2 is expressed in embryonic stem cells and germ cells and interacts with DAZ (Deleted in AZoospermia) and DAZ-like proteins.. Proc Natl Acad Sci U S A.

[29] Fox M, Urano J, Reijo Pera RA (2005). Identification and characterization of RNA sequences to which human PUMILIO-2 (PUM2) and deleted in Azoospermia-like (DAZL) bind.. Genomics.

[30] Nakahata S, Katsu Y, Mita K, Inoue K, Nagahama Y (2001). Biochemical identification of *Xenopus* Pumilio as a sequence-specific cyclin B1 mRNA-binding protein that physically interacts with a Nanos homolog, Xcat-2, and a cytoplasmic polyadenylation element-binding protein.. J Biol Chem.

[31] Gerber AP, Herschlag D, Brown PO (2004). Extensive association of functionally and cytotopically related mRNAs with Puf family RNA-binding proteins in yeast.. PLoS Biol.

[32] Cheong CG, Hall TM (2006). Engineering RNA sequence specificity of Pumilio repeats.. Proc Natl Acad Sci U S A.

[33] Opperman L, Hook B, DeFino M, Bernstein DS, Wickens M (2005). A single spacer nucleotide determines the specificities of two mRNA regulatory proteins.. Nat Struct Mol Biol.

[34] Sonoda J, Wharton RP (2001). *Drosophila* Brain Tumor is a translational repressor.. Genes Dev.

[35] Budnik V, Koh YH, Guan B, Hartmann B, Hough C (1996). Regulation of synapse structure and function by the *Drosophila* tumor suppressor gene *dlg.*. Neuron.

[36] Guan B, Hartmann B, Kho YH, Gorczyca M, Budnik V (1996). The *Drosophila* tumor suppressor gene, *dlg,* is involved in structural plasticity at a glutamatergic synapse.. Curr Biol.

[37] Chen K, Featherstone DE (2005). Discs-large (DLG) is clustered by presynaptic innervation and regulates postsynaptic glutamate receptor subunit composition in *Drosophila.*. BMC Biol.

[38] Baines RA (2005). Neuronal homeostasis through translational control.. Mol Neurobiol.

[39] Lawrence CE, Altschul SF, Boguski MS, Liu JS, Neuwald AF (1993). Detecting subtle sequence signals: A Gibbs sampling strategy for multiple alignment.. Science.

[40] Clarke PA (1999). Labeling and purification of RNA synthesized by in vitro transcription.. Methods Mol Biol.

[41] White EK, Moore-Jarrett T, Ruley HE (2001). PUM2, a novel murine puf protein, and its consensus RNA-binding site.. RNA.

