# Supplementary material for: Identification of Synaptic Targets of Drosophila Pumilio
Source: PLoS Comput Biol. 2008 Feb 29;4(2):e1000026. doi: 10.1371/journal.pcbi.1000026 (PMC2265480; doi:10.1371/journal.pcbi.1000026)
Supplement: Text S1 — Supplementary Material (0.05 MB DOC) [file pcbi.1000026.s001.doc]

Text S1

*Model evaluation with binding data*

The quantitative EMSA data provided us with an opportunity to evaluate the quality and accuracy of our three alternative models. From Table 1 we can see that a pattern match with NRE_PAT does not guarantee strong binding of Pum. On the other hand, two of the four sequences that strongly bind Pum lack a match to NRE_PAT. For NRE_M8 and NRE_M10, we calculated the correlation coefficient between the prediction scores and the binding affinities (Figure S1). For simplicity, the prediction score is the maximum matrix score of all the sites in a sequence. The correlation between the prediction scores of NRE_M10 and the Pum binding affinities is statistically significant and better than that of NRE_M8 (0.67 vs. 0.41). Therefore, our binding data suggest that NRE_M10 is more accurate than the other two models, supporting the assumption behind NRE_M10, i.e., only Box B is important for Pum binding.

*Conservation of Pum binding specificity between fly and mouse*

Cross-species comparison of PUF family protein sequences, from yeast to human, indicated that the RNA-binding domain of Pum is highly conserved, particularly in the RNA-interacting amino acid residues (Wang et al. 2002). This suggests that the Pum-binding sites in the mRNA should be conserved as well. To test this hypothesis, we obtained the mouse SELEX sequence data from White et al. (2001) and constructed matrix MmSelex_M8 (Figure S2A) using the same method as with NRE_M10. The prediction scores calculated with this matrix on our mRNA test sequences correlate remarkably well with the binding data with fly Pum (Figure S2B), suggesting that the binding specificities of the fly Pum and mouse PUM2 are very similar. Therefore, it is likely that the Pum binding specificity is conserved between *Drosophila* and mammals.

*Estimation of false positive predictions*

To estimate the false positive rate for each matrix, we randomly shuffled its columns, and then scored the sequences with the same method as the original matrix. When the matrix score at a position in a *D. melanogaster* sequence exceeds the threshold, we count it as a predicted site (a hit). The predicted site is considered conserved if it is aligned or overlaps with a predicted site in the corresponding *D. pseudoobscura* sequence. A (conserved) sequence hit is counted when a sequence contains at least one (conserved) predicted site. The mean and standard deviation of hits were calculated with 500 such shuffling tests. The average hit of shuffled matrices can serve as an estimation of the false positives. We performed the random shuffling tests with NRE_M10 and MmSelex_M8 on the 151 synaptic genes. The results are shown in Figure S3A and S3B, respectively. Quantitatively, the estimated false positive rate of sequence hit for NRE_M10 is 42.6% (without conservation filtering) and 27.7% (with conservation filtering). The corresponding estimations for MmSelex_M8 are 60.7% and 46.5%. We can see that using conservation information from a second fly species significantly reduces the false positive rate. In addition, the observed hits of our NRE/Pum-binding models are significantly higher than random hits (2.5 to >4 standard deviations above the mean), making us more confident that our predictions are likely to include a significant fraction of authentic binding sites.

Our results suggest that NRE_M10 and MmSelex_M8 are relatively good models for NRE or Pum-binding sites in *Drosophila*. Although the prediction scores of MmSelex_M8 fit the *in vitro* binding data even better than those of NRE_M10, each of the two motif models has its merits. Because NRE_M10 is constructed based on sequences of the natural Pum-binding sites, it may better reflect the *in vivo* binding property of Pum. On the other hand, NRE_M10 may be biased toward the pattern in *hb* and *bcd* NREs, which are dominant in our training data set. NRE_M10 is two bases longer than MmSelex_M8 and has higher total information content; thus, it appears to be more discriminative against false-positives, as shown in Figure S3. The major difference between NRE_M10 and MmSelex_M8 is the extra two positions at the 5' end of NRE_M10. These two positions are known to be important for Pum to recruit Nos, but not for Pum binding *per se* (Sonoda and Wharton 1999).

References

Sonoda, J. and Wharton, R.P. 1999. Recruitment of Nanos to hunchback mRNA by Pumilio. Genes Dev 13(20): 2704-2712.

Wang, X., McLachlan, J., Zamore, P.D., and Hall, T.M. 2002. Modular recognition of RNA by a human pumilio-homology domain. Cell 110(4): 501-512.

White, E.K., Moore-Jarrett, T., and Ruley, H.E. 2001. PUM2, a novel murine puf protein, and its consensus RNA-binding site. Rna 7(12): 1855-1866.

Figure Legends

Figure S1. Correlation between matrix prediction scores and Pum-binding affinities. The abscissa is the measured percentage binding of Pum to the mRNA target. The ordinate is the prediction score, which is the maximum matrix score of all the sites in a sequence. The 12 data points represent 12 mRNA sequences (nine test sequences in Table 1 and three control sequences). The Pearson correlation coefficient (cor) and its *p-value* are shown in the upper left corner. (A) Correlation for matrix NRE_M8. (B) Correlation for matrix NRE_M10.

Figure S2. (A) Base-frequency matrices obtained using Gibbs Sampler with mouse SELEX sequence data from White et al. (2001). Position 5 is a motif gap as in Gibbs output, which means that the base in this position is irrelevant. DNA notation is used as in Figure 2 of main text. (B) Correlation between matrix prediction scores and Pum-binding affinities for MmSelex_M8. Notations are the same as in Figure S1.

Figure S3. Estimation of false positives with random shuffle tests on the 151 synaptic genes. Shuffling times *n* = 500. (A) for matrix NRE_M10. (B) for matrix MmSelex_M8. The gray bars represent the hits with the original matrix. The black bars represent the average hits with randomly shuffled matrices. The error bar is the standard deviation across the 500 shuffling tests.

Figure S4. *dlg1* gene structure as shown in FlyBase Genome Browser. Transcript dlg1-RA and dlg1-RC are located on non-overlapping regions on the fly genome.

Figure S5. Comparison of the overlap of our Pum target predictions with the adult specific targets from Gerber et al. (2006) in the synaptic gene set. Pred+ and Pred- represent the number of our positive or negative prediction, respectively. PD+ and PD- represent the number of positive or negative pulled-down targets from Gerber et al. (2006), respectively.

Table S1. NRE_PAT predictions

Table S2. NRE_M8 predictions

Table S3. NRE_M10 predictions

Table S4. Segmentation pattern in embryos of modified hunchback gene transformant lines

Table S5. Synaptic gene list

Distinct 3’UTR sequences of the 151 synaptic genes of *D. melanogaster* and their alignment to putative *D. pseudoobscura* counterparts are included in file “synaptic_gene_aligned_3UTR.txt”. The 3'UTR sequences (including the stop codon) were determined with BDGP Release 3.1 annotation of *D. melanogaster*. The alignment of 3’UTR segments was obtained from whole-genome alignment between *D. melanogaster* and *D. pseudoobscura* produced by the BDGP at Lawrence Berkeley National Laboratory.
