# Supplementary figures and images for: Identification of Synaptic Targets of Drosophila Pumilio
Source: PLoS Comput Biol. 2008 Feb 29;4(2):e1000026. doi: 10.1371/journal.pcbi.1000026 (PMC2265480; doi:10.1371/journal.pcbi.1000026)

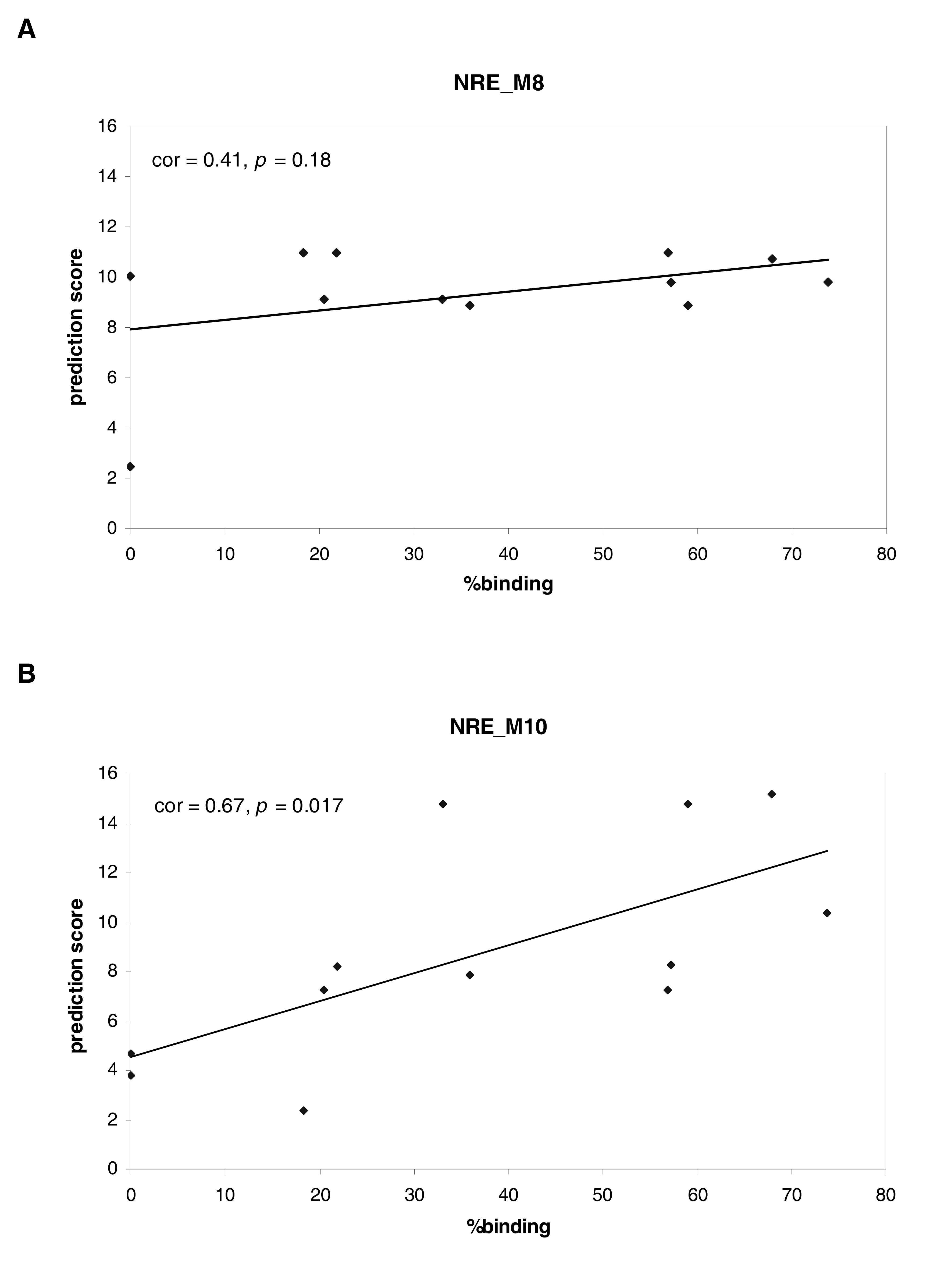

Supplement: Figure S1 — Correlation Between Matrix Prediction Scores and Pum-Binding Affinities. The abscissa is the measured percentage binding of Pum to the mRNA target. The ordinate is the prediction score, which is the maximum matrix score of all the sites in a sequence. The 12 data points represent 12 mRNA sequences (nine test sequences in Table 1 and three control sequences). The Pearson correlation coefficient (cor) and its p-value are shown in the upper left corner. (A) Correlation for matrix NRE_M8. (B) Correlation for matrix NRE_M10. (0.16 MB TIF) [file pcbi.1000026.s002.tif]

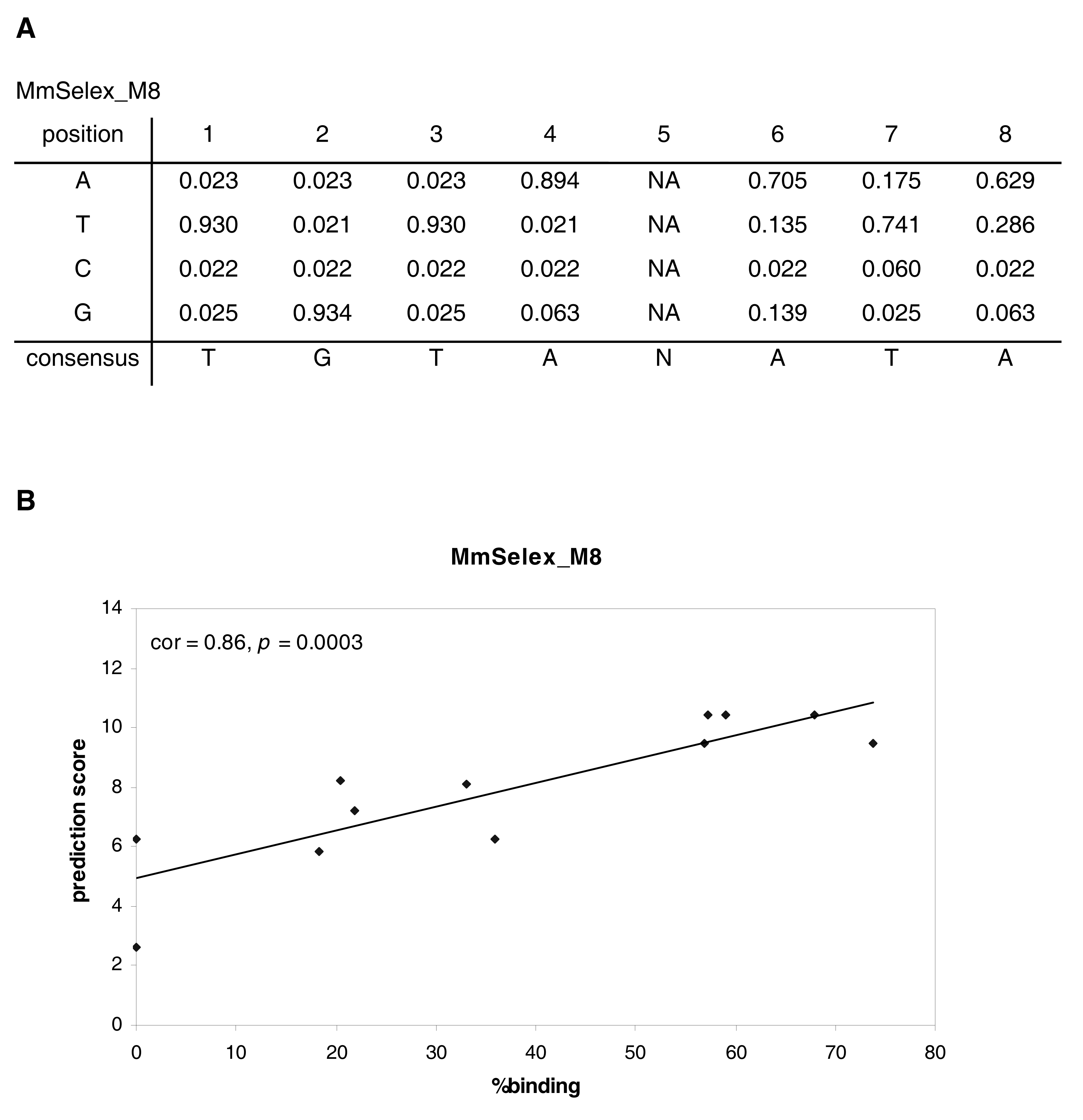

Supplement: Figure S2 — MmSelex_M8 Matrix and the Correlation of Its Prediction Scores to Pum-Binding Affinities. (A) Base-frequency matrices obtained using Gibbs Sampler with mouse SELEX sequence data from White et al. [41]. Position 5 is a motif gap as in Gibbs output, which means that the base in this position is irrelevant. DNA notation is used as in Figure 2. (B) Correlation between matrix prediction scores and Pum-binding affinities for MmSelex_M8. Notations are the same as in Figure S1. (0.19 MB TIF) [file pcbi.1000026.s003.tif]

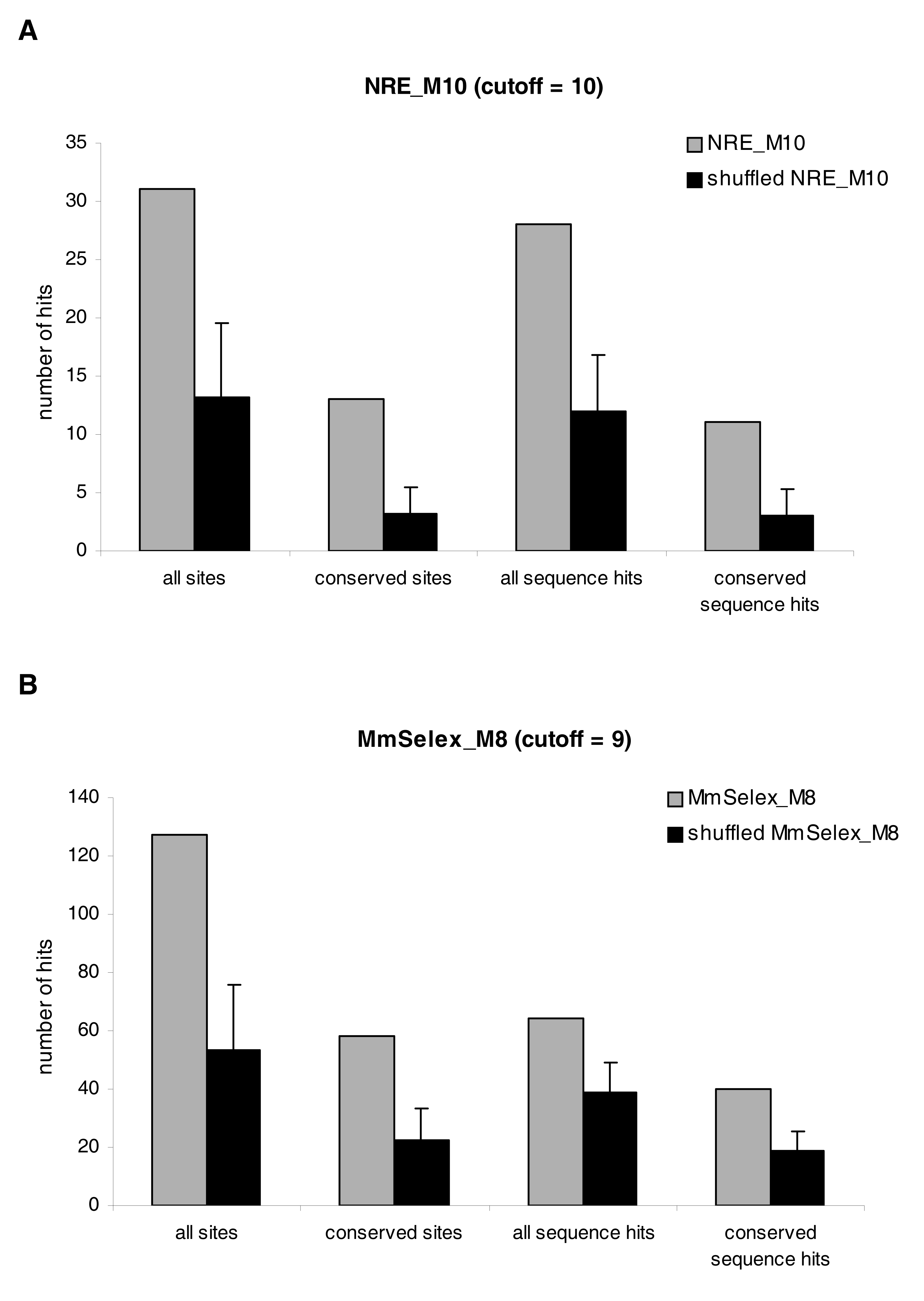

Supplement: Figure S3 — Estimation of False Positives with Random Shuffle Tests on the 151 Synaptic Genes. Shuffling times n = 500. (A) Matrix NRE_M10. (B) Matrix MmSelex_M8. The gray bars represent the hits with the original matrix. The black bars represent the average hits with randomly shuffled matrices. The error bar is the standard deviation across the 500 shuffling tests. (0.21 MB TIF) [file pcbi.1000026.s004.tif]

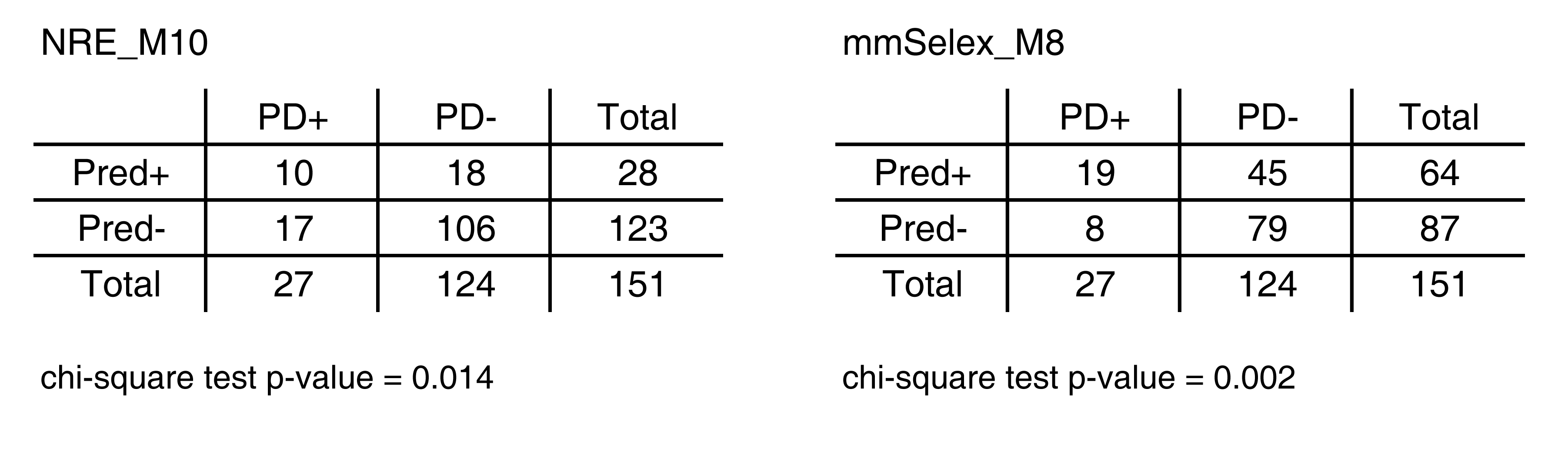

Supplement: Figure S5 — Comparison of the Overlap of Our Pum Target Predictions with the Adult Specific Targets from Gerber et al. [15] in the Synaptic Gene Set. Pred+ and Pred− represent the number of our positive or negative prediction, respectively. PD+ and PD− represent the number of positive or negative pulled-down targets from Gerber et al. (2006), respectively. (0.09 MB TIF) [file pcbi.1000026.s006.tif]
